# Hypomethylation of ETS Transcription Factor Binding Sites and Upregulation of PARP1 Expression in Endometrial Cancer

**DOI:** 10.1155/2013/946268

**Published:** 2013-05-13

**Authors:** Fang-Fang Bi, Da Li, Qing Yang

**Affiliations:** ^1^Department of Obstetrics and Gynecology, Shengjing Hospital, China Medical University, Shenyang 110004, China; ^2^Department of Physiology, Institute of Basic Medical Sciences, China Medical University, Shenyang 110001, China

## Abstract

Although PARP1 promoter methylation is involved in the regulation of PARP1 expression in human keratinocyte lines and lymphoblastoid cell lines, its roles in human endometrial cancer are unknown. DNA from forty normal endometrium (NE) and fifty endometrial adenocarcinoma (EAC) tissues were analyzed by bisulfite sequencing using primers focusing on the core promoter region of PARP1. Expression levels of PARP1 were assessed by immunohistochemistry and real-time PCR. Associations between patient clinicopathological characteristics and PARP1 protein levels were assessed by Fisher's exact test. Here, PARP1 mRNA and protein were overexpressed in EAC tissues (*P* < 0.05). CpG sites within the ETS motif in the PARP1 promoter exhibited significant hypomethylation in EAC tissues, and there was a significant negative correlation between PARP1 mRNA levels and the number of methylated sites in both NE and EAC tissues (*R* ^2^ = 0.262, *P* < 0.001). Notably, PARP1 protein expression was associated with FIGO stage (*P* = 0.026), histological grade (*P* = 0.002) , and body mass index (*P* = 0.04). Our findings imply that PARP1 overexpression may participate in endometrial cancer progression, and abnormal hypomethylation of CpG sites within the ETS motif in the core promoter region may be responsible for PARP1 overexpression in EAC tissues.

## 1. Introduction

Endometrial cancer (EC) is the most common gynecologic malignancy in the United States [1], but the molecular mechanisms involved in EC initiation and progression remain largely unknown. Considerable evidence suggests that progesterone and the progesterone receptor may in part be responsible for the pathogenesis of EC by antagonizing estrogen-driven endometrial proliferation [2]. Interestingly, several studies have indicated that poly-(ADP-ribose) polymerase 1 (PARP1), which is a pivotal single-stranded DNA break repair gene, can interact with the DNA binding domain of progesterone receptor. Meanwhile, the expression of PARP1 gradually increases in each stage of EC, which is highly correlated with progesterone receptor levels [3]. Therefore, it is conceivable that PARP1 plays an important role in the development of EC through its involvement in regulating progesterone receptor expression. Recent studies have suggested that PARP1 promoter methylation is involved in the regulation of PARP1 expression in human keratinocyte lines [4] and lymphoblastoid cell lines [5]. However, little is known about the transcriptional regulation of PARP1 in EC. Our present study is the first to analyze DNA methylation patterns in the core promoter region of PARP1, showing that the abnormal methylation patterns, especially around the E26 transformation-specific (ETS) motif, may be responsible for PARP1 overexpression in EC. Moreover, correlation of PARP1 expression with clinicopathological characteristics was analyzed.

## 2. Materials and Methods

### 2.1. Patients and Tissues Collection

The present study was approved by the Institutional Review Board at China Medical University, and all the participants gave informed consent. Forty normal endometrium (NE) tissues (mean age, 52.73 ± 10.88) and fifty endometrial adenocarcinoma (EAC) tissues (mean age, 54.17 ± 10.09) were collected from Shengjing Hospital of China Medical University between 2010 and 2012. All tissues were obtained at the time of primary surgery before any chemotherapy or radiotherapy and were examined by hematoxylin and eosin staining. NE tissues were collected from hysterectomies for prolapse of uterus or CIN III patients, including 28 proliferative endometrium and 12 secretory endometrium. EAC tissues were staged according to the International Federation of Gynecology and Obstetrics (FIGO 2009) by three experienced pathologists. The fifty EAC tissues were divided into 29 cases of stage I, 6 cases of stage II, and 15 cases of stage III.

### 2.2. Real-Time Quantitative PCR

Total RNA was extracted from the NE and EAC tissues using Trizol reagents (Invitrogen, Carlsbad, CA, USA) according to a standard protocol. Then, RNA was reverse transcribed using the PrimeScript RT Master Mix Kit (TaKaRa, Dalian, China) and amplified by SYBR Premix Ex TaqTM II (TaKaRa) using an ABI 7500 instrument (Applied Biosystems, Foster City, CA, USA). The GAPDH gene was used as an internal control for normalization. The primer sequences were as follows: PARP1: 5′-GAGTCGGCGATCTTGGACC-3′ (F) and 5′-TGACCCGAGCATTCCTCG-3′ (R); GAPDH: 5′-AGGTGAAGGTCGGAGTCA-3′ (F) and 5′-GGTCATTGATGGCAACAA-3′ (R). The PCR conditions were as follows: 45 cycles of 95°C for 10 s and 60°C for 20 s. Relative mRNA expression was analyzed in triplicate using the 2^−ΔΔCT^ method.

### 2.3. Immunohistochemistry

Immunohistochemistry was performed with the biotin-streptavidin peroxidase complex method. Briefly, serial 4-*μ*m sections were dewaxed in xylene and rehydrated in graded alcohol. After microwave antigen retrieval, the sections were incubated overnight at 4°C with mouse monoclonal anti-PARP1 antibody (1 : 100; Santa, Cruz Biotechnologies, USA). 3,3′-Diaminobenzidine (DAB) was used as the chromogen. Nuclei were counterstained with hematoxylin and examined with a light microscope at a magnification of 100*x* and 400*x*. All cases were analyzed by two independent pathologists, blinded to the clinical data. The level of staining was classified into four groups by scoring the percentage of positive cells: 0, negatively stained cells; 1, <33% of cells stained; 2, 33 to 66% of cells stained; 3, >66% of cells stained.

### 2.4. Bisulfite Sequencing

Genomic DNA was extracted from NE and EAC tissues using a TIANamp Genomic DNA Kit (Tiangen Biotech, Beijing, China). Sodium bisulfite conversion was performed using the EZ DNA Methylation-Direct kit (Zymo research, Orange, USA) according to the manufacturer's protocol. PCR amplification was performed using the special Hot-Start DNA polymerase (ZymoTaq Premix, Zymo Research) with the following reaction conditions: 95°C for 2 min; 40 cycles of 95°C for 30 s, 56°C for 30 s, and 72°C for 45 s; then 72°C for 7 min. Two pairs of primers were used: round I, 5′-TTGGGATAGAATAATTAAAG-3′ (F) and 5′-AACTTTTCCTACAACATCAA-3′ (R); round II, 5′-TAGAATAATTAAAGGGGTGG-3′ (F) and R: 5′-ACAACATCAACAAAACCTT-3′ (R). The PCR fragment was ligated into a pMD18-T Vector (TaKaRa), and the recombinant plasmid was transformed into JM109 cells (TaKaRa). Ten positive clones of each sample were selected and sequenced on an ABI 3730 automated sequencer.

### 2.5. Statistical Analysis

Regression analysis was used to examine the possible relationship between PARP1 mRNA levels and the number of methylated CpG sites within the ETS motif. The association between patient clinicopathological characteristics and PARP1 protein levels was determined using Fisher's exact test. The data are presented as mean ± SE. Statistical differences were evaluated by unpaired Student's*t*-test using SPSS v11.5 software (SPSS Inc., Chicago, IL, USA). Statistical significance was set at *P* < 0.05.

## 3. Results

### 3.1. PARP1 Expression Was Upregulated in EAC Tissues

**Figure 1:**
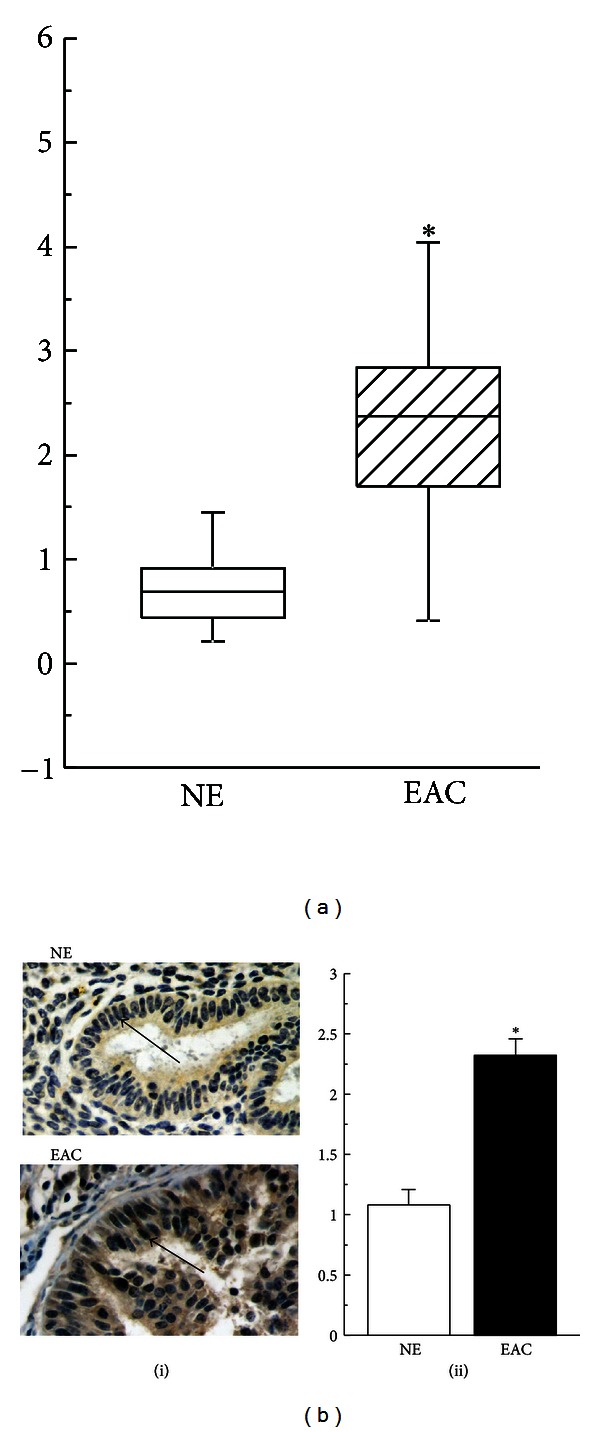
Overexpression of PARP1 protein and mRNA in EAC tissues. (a) Quantification of relative PARP1 mRNA levels. (b(i)) Sections were subjected to immunostaining for PARP1. Arrow denotes positive staining for PARP1 in the nuclei. (b(ii)) Summary of scoring the percentage of positive cells from the measurements shown in (b(i)). Bar graphs show mean ± SE. *P < 0.05 versus NE. Magnification is 400*x*. NE, normal endometrium; EAC, endometrial adenocarcinoma.

Real-time PCR and immunohistochemical analysis showed that PARP1 mRNA and protein were overexpressed in EAC tissues compared to NE tissues (*P* < 0.05; Figures 1(a) and 1(b)).

### 3.2. Correlation of PARP1 Expression with Clinicopathological Characteristics in EAC Tissues

The associations between PARP1 expression and clinicopathological features are shown in Table 1. PARP1 expression was associated with FIGO stage (*P* = 0.026), histological grade (*P* = 0.002), and body mass index (*P* = 0.04). No significant associations were observed between expression of PARP1 and age, myometrial invasion, or lymph node metastasis.

### 3.3. EAC Tissues Displayed a Hypomethylated ETS Motif in the Core Promoter Region of PARP1

**Figure 2:**
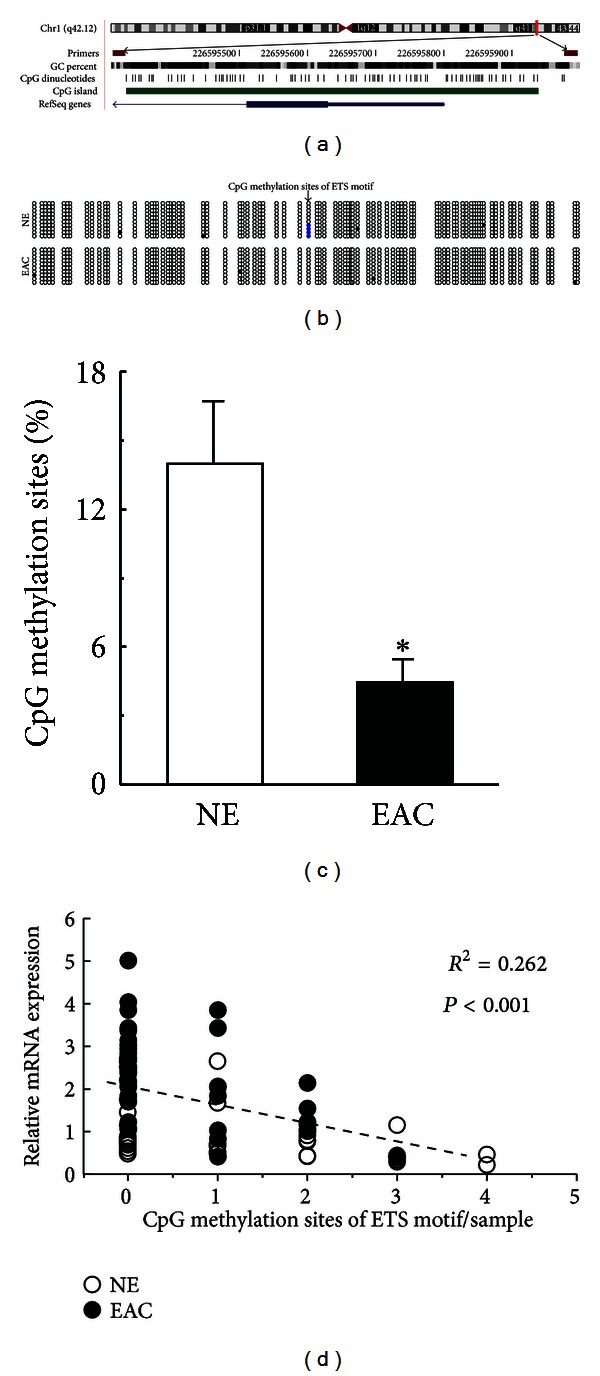
Hypomethylation of the ETS transcription factor binding sites in the promoter of PARP1 in EAC tissues. (a) Location of PARP1 core promoter CpG sites. Genomic coordinates are shown, along with the primer-amplified fragments, GC percentage, location of individual CpG dinucleotides (dashes), CpG island (green bar), and the PARP1 RefSeq gene (exon 1 shown as a blue box and intron shown as an arrowed line). The arrow indicates the transcriptional direction. (b) Changes in methylation patterns in the core promoter region of PARP1. The circles correspond to CpG sites denoted by the black dashes in (a). Closed circles, methylation; open circles, unmethylation. Ten individual clones were sequenced for each sample. (c) Summary of the methylated sites in a CpG within the ETS motif in NE and EAC tissues. Bar graphs show mean ± SE. *P < 0.05 versus NE. (d) Correlation between the relative PARP1 mRNA levels and the number of methylated sites in a CpG within the ETS motif for each sample. Open circles, NE; closed circles, EAC; NE, normal endometrium; EAC, endometrial adenocarcinoma.

To investigate the epigenetic mechanism responsible for PARP1 transcription, the DNA methylation status of the PARP1 core promoter region (from −190 to +496, +1 is the transcription initiation site) was determined in NE and EAC tissues by sequencing of PCR products from bisulfite-converted genomic DNA. As shown in Figures 2(b) and 2(c), CpG sites within the ETS motif exhibited significant hypomethylation in EAC tissues, compared to NE tissues (*P* < 0.05). In addition, we noted a significant negative correlation between PARP1 mRNA levels and the number of methylated sites within the ETS motif in both NE and EAC tissues (*R* ^2^ = 0.262, *P* < 0.001; Figure 2(d)).

## 4. Discussion

DNA methylation is an epigenetic modification involved in controlling gene transcription through interference with transcription factors binding to DNA [6]. ETS proteins are a family of evolutionary-related transcription factors that are widely distributed in the promoter of PARP1, which is responsible for the specific recognition of a common sequence motif, 5′-(C/A)GGA (A/T)-3′ [7], and promotes the assembly of transcriptional machinery [8]. The main findings of the present study are that EAC tissues have a relatively hypomethylated region of CpG sites around the ETS motif, and PARP1 mRNA levels show negative correlation with the number of methylated sites in both NE and EAC tissues. In addition, the available data suggest that ETS transcription factors play an important role in regulating PARP1 expression [8]. Therefore, there is preliminary evidence to suggest that increased PARP1 expression may be relevant to the abnormal methylation of the ETS motif in EAC tissues. Clinicopathological data indicated that PARP1 expression was significantly associated with high-grade tumors (*P* = 0.002) and early-stage tumors (*P* = 0.026). Notably, patients with high expression levels of PARP1 had a high body mass index. Similarly, Bai et al. suggested that PARP1^−/−^ mice showed reduced fat accumulation, higher energy expenditure, enhanced glucose oxidation, and protection against diet-induced obesity and insulin resistance [9]. As is previously known, obesity is a possible risk factor for EC, but the underlying mechanisms are poorly understood [10]. Our study shed new insight into the observed obesity-EC associations: it is proposed that obesity may be an epiphenomenon underlying abnormal PARP1 metabolism rather than the direct cause of the EC. However, the relationship between obesity and EC is complex, and there are many other factors involved.

## 5. Conclusions

These data suggest a specific role for the hypomethylated ETS motif-mediated PARP1 overexpression in EC progression, but a clear molecular model regarding how the abnormal methylation of the ETS motif affects the transcription of PARP1 needs to be established by further experimental evidence. If we can clarify the transcriptional mechanism of PARP1, a more specific epigenetic therapy could be developed for EC in the future. 

## Figures and Tables

**Table 1:** Association of PARP1 expression in 50 EAC tissues with clinicopathological features.

Factors	PARP1 immunoreactivity score			*P*
	*n*	<2	≥2	
Age				
<60	38	9	29	
≥60	12	4	8	*P* = 0.71
Tumor FIGO stage				
I	29	4	25	
II or III	21	9	12	*P* = 0.027
Histological grade				
G_1_	19	10	9	
G_2_ or G_3_	31	3	28	*P* = 0.002
Myometrial invasion				
<1/2	32	7	25	
≥1/2	18	6	12	*P* = 0.504
Lymph node metastasis				
Negative	33	8	25	
Positive	17	5	12	*P* = 0.74
Body mass index				
<25	29	11	18	
≥25	21	2	19	*P* = 0.047
